# Chemotaxis of bio-hybrid multiple bacteria-driven microswimmers

**DOI:** 10.1038/srep32135

**Published:** 2016-08-24

**Authors:** Jiang Zhuang, Metin Sitti

**Affiliations:** 1 Department of Mechanical Engineering, Carnegie Mellon University, Pittsburgh, PA 15213, USA; 2 Physical Intelligence Department, Max Planck Institute for Intelligent Systems, 70569 Stuttgart, Germany

## Abstract

In this study, in a bio-hybrid microswimmer system driven by multiple *Serratia marcescens* bacteria, we quantify the chemotactic drift of a large number of microswimmers towards L-serine and elucidate the associated collective chemotaxis behavior by statistical analysis of over a thousand swimming trajectories of the microswimmers. The results show that the microswimmers have a strong heading preference for moving up the L-serine gradient, while their speed does not change considerably when moving up and down the gradient; therefore, the heading bias constitutes the major factor that produces the chemotactic drift. The heading direction of a microswimmer is found to be significantly more persistent when it moves up the L-serine gradient than when it travels down the gradient; this effect causes the apparent heading preference of the microswimmers and is the crucial reason that enables the seemingly cooperative chemotaxis of multiple bacteria on a microswimmer. In addition, we find that their chemotactic drift velocity increases superquadratically with their mean swimming speed, suggesting that chemotaxis of bio-hybrid microsystems can be enhanced by designing and building faster microswimmers. Such bio-hybrid microswimmers with chemotactic steering capability may find future applications in targeted drug delivery, bioengineering, and lab-on-a-chip devices.

Bio-hybrid microsystems, which integrate swimming bacteria^1^,^2^,^3^,^4^,^5^,^6^,^7^,^8^,^9^,^10^,^11^,^12^,^13^,^14^,^15^,^16^, algae^17^, or contractile cells^18^,^19^,^20^,^21^ with synthetic functional components, has the potential of overcoming the grand challenges in miniaturizing on-board actuation and power supply for microsystems. Prototypes, including microswimmers^3^,^5^,^10^,^12^,^13^,^14^,^15^,^16^,^17^, micromotors^4^,^8^,^9^,^11^, and microfluidic components^1^,^2^,^6^,^7^, have been extensively developed in the last decade for potential applications in medicine, bioengineering, and lab-on-a-chip devices. Microswimmers are of particular interest for use in future biomedical applications such as targeted drug delivery in stagnant liquid media^22^,^23^. To bias the otherwise stochastic motion of bio-hybrid microswimmers, various external physical fields, such as magnetic and electric fields, have been applied^24^,^25^,^26^. However, the control has only been demonstrated for single microswimmers and requires complex external equipment to generate and deliver the required physical fields. Therefore, this study aims to use biological cell sensory, specifically chemotactic, response and environmental stimuli to steer a large number (swarm) of microswimmers without needing any external equipment.

Flagellated bacteria, like *Serratia marcescens* (*S. marcescens*), is one of the significant candidates for the actuators of self-propelled bio-hybrid microswimmers, not only because of their ease of cultivation and high motility, but also due to their chemotactic behavior. The chemotactic behavior is a potential elegant way to control the microswimmers at the swarm level for future targeted drug delivery purposes, because chemical gradients are ubiquitous in human body. Although several studies have observed chemotaxis of the multi-bacteria-driven microswimmers^27^,^28^,^29^,^30^, none of them has characterized the physical driving mechanisms and important factors of the associated biased random walk and the importance of microswimmers’ motility for chemotaxis. pH-taxis in bio-hybrid microsystems has been studied recently^31^, however, bacterial sensing mechanism of pH is fundamentally different with that of canonical chemotaxis: pH-taxis is mediated by cytoplasmic pH level^32^, while canonical chemoattractants and chemorepellants are sensed by transmembrane chemoreceptors^33^. As a result, the physical driving mechanisms of the collective chemotaxis among multiple bacteria attached to a microswimmer has been unclear to date. Despite the well established theory on bacterial chemotaxis^34^,^35^, it is not readily understandable how the microswimmer, consisting of a microstructure propelled by multiple randomly attached bacteria, is endowed with chemotaxis. To shed light on this, we characterized the chemotaxis in a multi-bacteria-driven microswimmer system in a fashion helpful to understand the associated physical mechanisms and meaningful to develop bio-hybrid microswimmers with enhanced chemotactic behavior.

Relying on a precise microfluidic chemical gradient generator, we first traced out the chemotactic response of the free swimming bacteria *S. marcescens* to L-serine (chemoattractant), and an optimal concentration gradient that leads to the strongest chemotactic response was empirically determined. Using the optimal gradient, chemotactic drifting process of multi-bacteria-driven microswimmer swarms were imaged and quantified. Finally, by tracking the individual microswimmers and statistically analyzing the trajectories, we identified the critical factors and the behind physical mechanisms which enabled the chemotaxis in the multi-bacteria-driven microswimmers.

## Results

**Figure 1:**
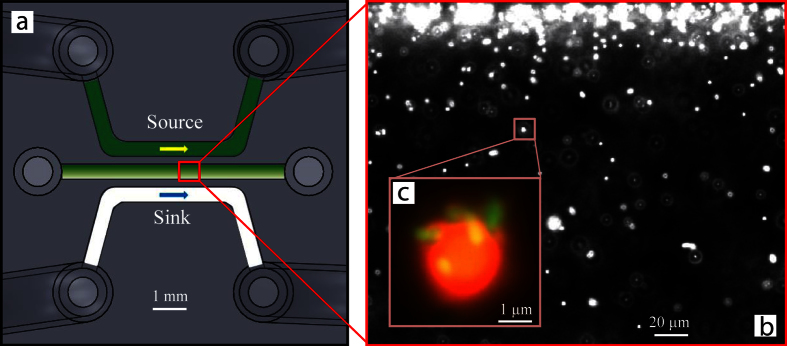
Three-channel microfluidic concentration gradient generator and the bio-hybrid bacteria-driven microswimmers. (**a**) *Top view* of the three parallel microfluidic channels, where the green color (source channel) indicates a nonzero concentration of the chemoattractant while the white (sink channel) stands for a concentration of zero; the concentrations in the source and sink channels were maintained by flowing fluid through the channels at a constant rate. At steady state, a linear concentration profile of the chemoattractant is established in the sample (middle) channel. The dimensions of the cross-section of each channel are 500 *μ*m × 200 *μ*m (width × height). (**b**) Fluorescent image of a swarm of microswimmers. The entire width of the channel is fully captured in the image. The bright dots in the field indicate individual microswimmers and the bigger bright areas closer to the source channel correspond to clusters of microswimmers that have accumulated as a result of chemotactic drift. (**c**) Fluorescent image of a sample microswimmer, which is composed of a 3.1 *μ*m diameter polystyrene bead (fluorescent red) and several randomly attached *S. marcescens* bacteria (yellow-green).

In the middle sample channel of a microfluidic chemical gradient generation device (Fig. 1(a)), we created a quiescent fluid environment with a spatial concentration gradient of L-serine to characterize the chemotaxis of the bio-hybrid microswimmers. The bio-hybrid microswimmers were synthesized by attaching multiple flagellated bacteria *S. marcescens* to 3.1 *μ*m diameter polystyrene beads (see details in Methods). Based on the protocol, the mean number of bacteria assembled to a sample microswimmer was measured to be 9 with a standard deviation of 3.4 31. The position and orientation of the attached bacteria on the microbeads were purely random, resulting in high stochasticity of the configuration of bacteria on the bio-hybrid microswimmer (Fig. 1(c)). To achieve considerable chemotactic drift in such a system, we first characterized the chemotactic response of the bioactuator alone, *S. marcescens*, towards the chemoattractant L-serine. This step served to find the optimum concentration profile with the strongest chemotactic response to be applied to study the chemotaxis of the microswimmers.

### Bacterial chemotactic response to L-serine

**Figure 2:**
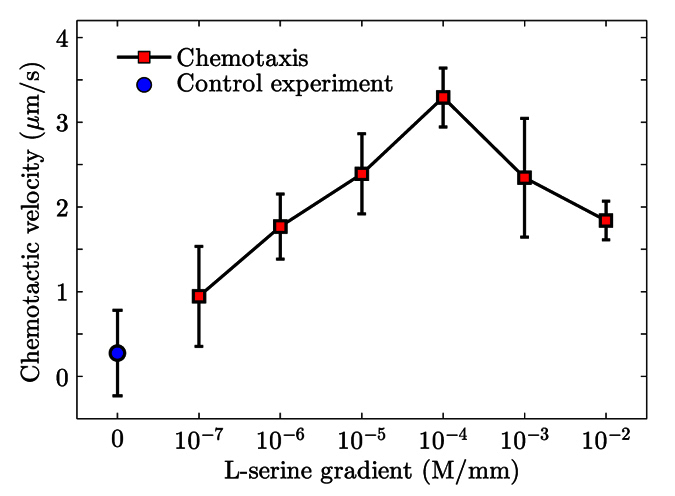
Chemotactic response of *S. marcescens* to a concentration gradient of L-serine. At the data points of chemotaxis (red squares), the corresponding L-serine gradients were created by a nonzero concentration in the source channel and a zero concentration (buffer flow) in the sink; the gradient of control sample (blue circle) was zero, enabled by simply inputing both source and sink with a buffer flow. For each concentration gradient, the average chemotactic velocity, *V*_*C*_, and its standard deviation (indicated by the error bar) were measured over five independent video samples which were taken from different locations in the sample channel (far away from any walls to avoid wall effects). The number of swimming trajectories captured in each video varied from 500 to 3000.

L-serine is a canonical and potent chemoattractant for bacteria like *E. coli* and *Salmonella typhimurium*^13^,^34^,^36^,^37^,^38^,^39^,^40^, and has shown to be mainly sensed by the abundant transmembrane receptor *Tsr*^41^,^42^,^43^. *S. marcescens*, a species that highly resembles *E. coli* in terms of motility and chemotaxis^44^,^45^, should exhibit remarkable chemotaxis to L-serine as well. It has been established that flagellated bacteria, such as *S. marcescens* and *E. coli*, swim through a combination of runs and tumbles, which are responsible for translation and random reorientation of the bacteria, respectively. The running state associates with counterclockwise rotation (CCW) of bacterial flagella, while the tumble state corresponds to clockwise (CW) rotation of flagella. In an environment with a chemoattractant gradient, bacteria decrease their tumble rate when they move towards a favorable direction while maintaining at a normal state (i.e. normal tumble rate) when moving towards the unfavorable direction. Such a biased tumble rate on individuals produces a population-level drift up the concentration gradient of the chemoattractant, which is described by the *chemotactic velocity*, *V*_*C*_^38^,^46^, as follows


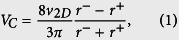


where *r*^+^ and *r*^−^ are the mean tumble rates when bacteria travel up and down the chemical gradient, respectively, and *v*_2*D*_ is the two-dimensional (2D) mean swimming speed of bacteria. Here, we tested the the chemotaxis of *S. marcescens* under a series of linear concentration profiles of L-serine, and quantified the chemotactic response using *V*_*C*_, which can be readily determined from the 2D trajectories of the swimming bacteria^45^. As shown in Fig. 2, the chemotactic velocity peaks around a concentration gradient of 10^−4^ M/mm, which is of the same order of magnitude with that of *E. coli*^39^; moreover, the chemotactic response trend is similar to that of *E. coli*, which suggests a resemblance in the signaling pathway dynamics for L-serine chemotaxis between these two bacterial species. Notice that increasing the concentration gradient also increases the average concentration of L-serine, the chemotactic velocity decreases at higher gradients due to receptor saturation kinetics at high ligand concentrations^39^,^45^.

### Chemotactic drift of microswimmers

**Figure 3:**
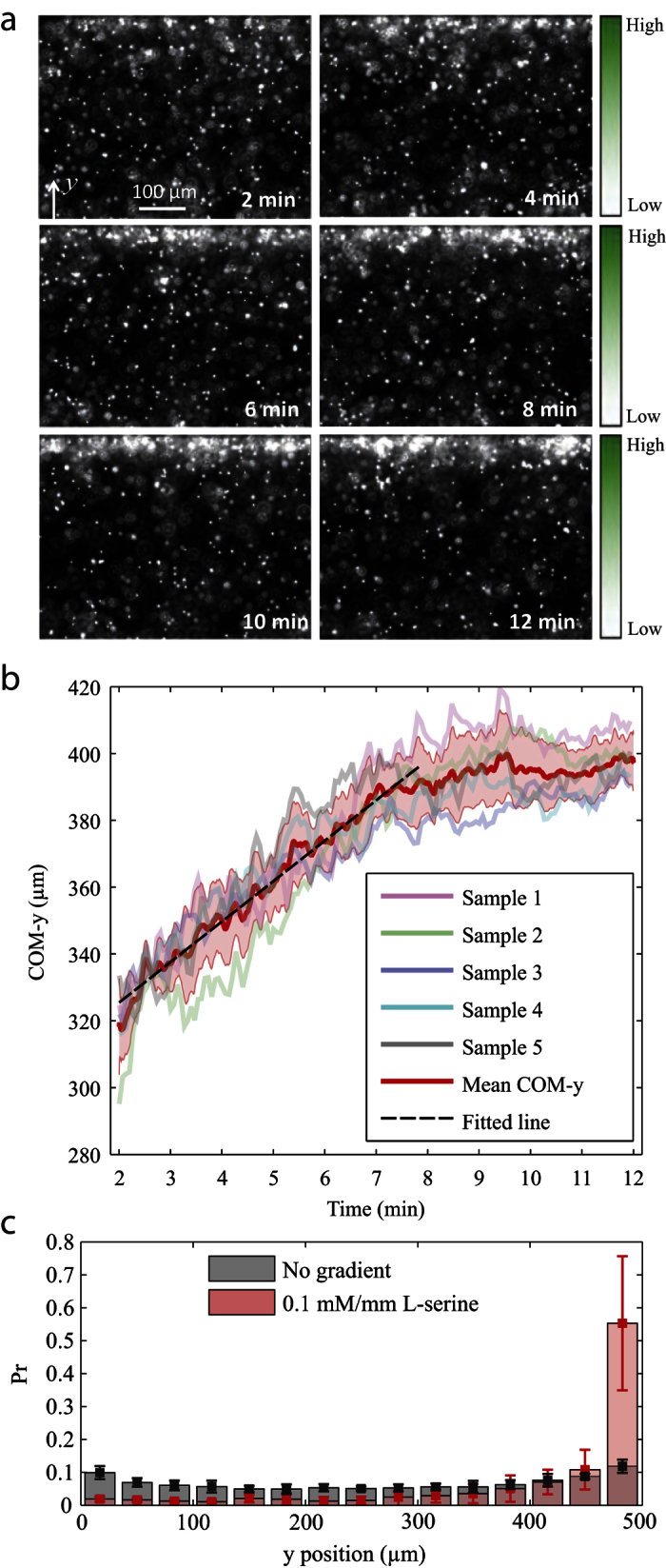
Chemotactic drift of the microswimmer swarm. (**a**) Fluorescent images show the distribution of microswimmers at a fixed location in the sample channel over time, of which the starting point is when the diffusion of the chemoattractant starts, i.e., when the flow is initiated in the source and sink channels. As indicated by the gradient color bars, the chemoattractant gradient is aligned with the *y*-dimension of the images and is along the width of the sample channel. The initial two minutes were not recorded in order to allow for disturbance-induced flows to settle down. (**b**) COM-*y* of the microswimmer swarm over time. For each of the five samples, 3,000 image frames were captured over 10 min and the COM-*y* of each frame is plotted. The red curve and the shaded area indicate the mean and standard deviation found among the five samples. (**c**) Probability distribution of microswimmers across the width of the sample channel at final state. The means and standard errors of the chemotaxis group (0.1 mM/mm gradient) were evaluated on five different samples while the control group (no gradient) was based on three independent samples.

**Figure 4:**
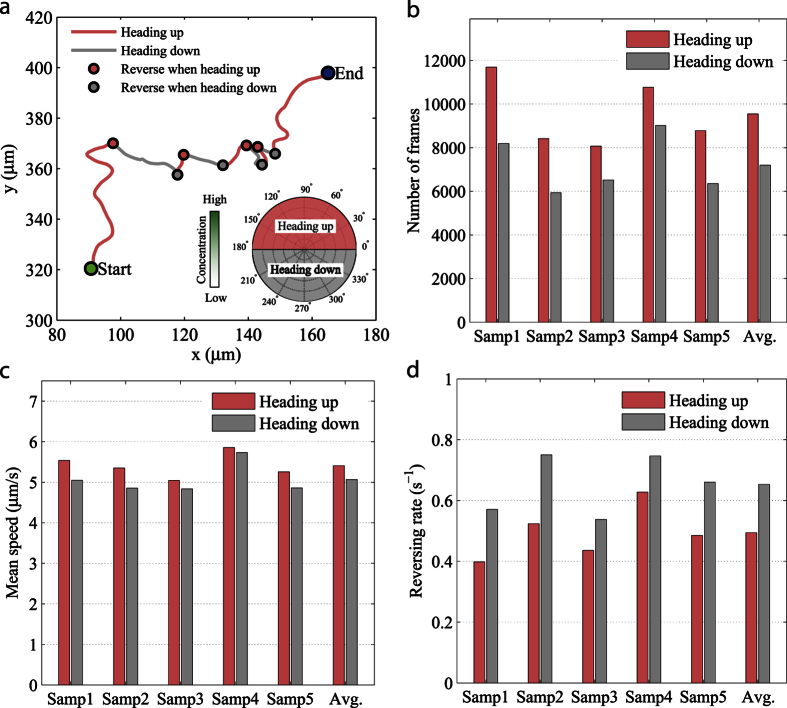
Trajectory analysis of chemotactic swimming in microswimmers. (**a**) A sample trajectory is decomposed into two kinds of segments: the heading-up, during which the microswimmer persistently moves up (+*y*) the L-serine gradient, and the heading-down, during which the microswimmer persistently moves down (−*y*) the gradient. Correspondingly, along the trajectory, there are two types of direction reversing along the *y*-axis: reverse when heading up (+*y* → −*y*) and reverse when heading down (−*y* → +*y*). (**b**) Across all trajectories captured in each sample, number of frames (corresponding to time duration) counted for the heading-up and heading-down segments. (**c**) The mean speeds of the heading-up segments and the heading-down segments extracted from all trajectories captured in each sample. (**d**) The direction reversing rates computed for the heading-up segments and the heading-down segments based on all the trajectories in each sample. More than 250 trajectories were captured for each of the five independent experiments (samples). The last two bars (Avg.) in each figure (**b**–**d**) show the average of the corresponding quantity over all samples.

After finding the optiumum concentration profile with the free swimming bacteria, we characterized the chemotaxis of bacteria-driven microswimmers. A total of five independent experiments were conducted to assess the consistency of the results; the microswimmers used in each experiment were assembled from an independent culture of bacteria. The sample numbers in Figs 3 and 4 identify the corresponding experiments from which the data were extracted.

As shown in Fig. 3(a), the initial uniform distribution of microswimmers in the sample channel evolved gradually into a highly biased distribution: the side with a higher concentration of L-serine was associated with a remarkably higher density of microswimmers than the other side. This indicates that the bio-hybrid microswimmer preserved the chemotactic behavior observed in the free swimming bacteria of *S. marcescens*. To quantify the apparent chemotactic drift of the microswimmer swarm, we examined the center of mass (COM) position of the microswimmers captured in each imaging frame and plotted its *y* component (COM-*y*) over time (Fig. 3(b)). The COM-*y* of a frame can be calculated by: COM-*y* = 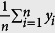, where *y*_*i*_ is the *y*-position of the *i*-th microswimmer, and *n* is the number of all the captured microswimmers. As can be seen in Fig. 3(b), the initial drift process (up to 7.5 min) traces out a linearly increasing COM-*y* over time, which suggests an approximately constant chemotactic drift velocity of the swarm. After the linear region, the distribution tends to stabilize to a *final state*. We observed that the motile microswimmers drifted to the higher concentration side of the sample channel and then formed clusters, whereas the microswimmers that remained scattered were non-motile, typically without bacteria attached. The clusters initially appeared because the motion of the microswimmers were constrained by the walls of the sample channel. Since both groups, the clusters and the scattered individuals, had rather low motilities, the system reached a relatively stable final state. Figure 3(c) presents the probability distributions of the *y*-position of the microswimmers for both the chemotactic samples at final state and the control samples at steady state. By a comparison between the two distributions, it can be concluded that the unidirectional drift of the microswimmers was due to chemotaxis instead of other factors, such as wall effects.

### Physical mechanisms of chemotaxis in microswimmers

Drift analysis based on swarm distributions hides important information about the physical driving mechanism of the individual microswimmers that enables the chemotactic behavior. In addition, calculating the chemotactic velociy from the COM-*y* leads to an underestimated value, largely due to the restricted motion of the microswimmers near the channel wall and the contribution from the non-motile microswimmers. In light of these limitations, we tracked the swimming trajectories of individual microswimmers and performed statistical analysis on the trajectories, aiming to unveil the physical mechanisms that drive the chemotaxis of individual microswimmers and thereby resulting the population level drift.

In the microfluidic device, the microswimmers were subject only to chemotactic stimulus along one dimension, namely along the *y*-axis; thus, the *x*- and *z*-components of the motion should be independent of the direction along the respective axis. In other words, the motion of the microswimmers was only biased along the *y*-axis, and hence a 1D model is sufficient to capture the chemotaxis of individual microswimmers. Developed in ref. 31, the drift velocity of a particle conducting 1D (along *y*-axis) random walk is given as


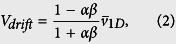


where *α* = *v*_−*y*_/*v*_+*y*_, is the speed ratio, i.e., the ratio of the mean speeds in the −*y* and +*y* directions, *β* = *t*_−*y*_/*t*_+*y*_, is the heading ratio, i.e., the ratio of the time spent moving towards −*y* and +*y* directions, and 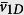 is the 1D mean speed, which in our case is the mean speed along the *y*-axis.

The 2D trace of a sample microswimmer can be decomposed into segments persistently heading towards +*y* (heading up) and segments persistently heading towards −*y* (heading down) based on the *y*-component of its instantaneous heading direction, as illustrated in Fig. 4(a). Therefore, the motion of the microswimmer along the *y*-axis is identical to a 1D biased random walk, and we can easily conform the trajectory analysis to the above model. Figure 4(b) shows the time spent moving along each direction of the *y*-axis, represented by the number of frames counted in each direction. Across five independent samples, the portion of time spent moving up the L-serine gradient is considerably higher than that spent traveling down the gradient, which we call a “heading bias” in this study. This suggests that individual microswimmers under the L-serine gradient exhibit a strong heading preference for moving up the gradient. A consistent heading bias is also found on the free-swimming bacteria (Fig. S1(a) in the supplementary material), but it is less pronounced than that revealed on the bio-hybrid microswimmers; this discrepancy is due to the prominent wall effect on the motion of free-swimming bacteria, where their natural chemotactic transport is constrained by the channel wall.

Another factor that contributes to the 1D drift velocity is the speed difference, revealed by the speed ratio, *α* in Eq. 2. As shown in Fig. 4(c), a measurable mean speed difference exists between the components of motion heading up and heading down, which we refer to as the speed bias. The heading-up component has a slightly higher mean speed, and the trend is consistent over all the samples. The free swimming bacteria also manifests a slight speed bias during chemotaxis in a similar fashion (Fig. S1(b) in the supplementary material). Since the translational speed is linearly related to the net propulsive force in the Stokes flow regime, the attached bacteria exert a slightly larger force when the microswimmer moves up the L-serine gradient; presumably, the propulsive force is biased due to a lower probability of flagellar clockwise rotation in the attached bacteria when the microswimmer swims up the gradient. The analysis based on swimming traces of motile microswimmers which were free from wall effects yielded a chemotactic drift velocity of 39.6 ± 12.9 *μ*m/min, to which the heading bias contributes nearly five times more than the speed bias does based on the relative significance of the heading ratio and the speed ratio. Thus, the heading bias is the dominant driving factor of the chemotactic drift in the bio-hybrid microswimmers.

To understand how the heading bias was generated in the bio-hybrid microswimmers, we further inspected the relationship between the *y*-direction reversing rate and the heading direction. As illustrated by Fig. 4(a), the *y*-direction reversing events along a sample swimming trace were classified into two types; reverses associated with the heading-up segments (+*y* → −*y*), and reverses associated with the heading-down segments (−*y* → +*y*). The heading-up reversing rate is defined as the total number of direction reverses occurring while moving up the gradient divided by the total length of time traveling in the upward direction, and vice versa for the heading-down reversing rate. Figure 4(d) shows the heading-up and heading-down reversing rates of each sample; the reversing rates when heading up are unanimously lower than those of heading down across the five samples. This concludes that, compared to moving down the gradient, the microswimmers showed higher persistence in their *y*-direction heading when moving up the L-serine gradient. Given that the attachment between bacteria and the microbeads is merely a physical adhesion^44^, the signaling transduction pathway of the bacteria should not be distorted; therefore, the probability of flagellar CW rotation of the attached bacteria is expected to be higher when the microswimmer moves down the gradient. The biased reversing rate of the microswimmers leads to the conjecture that the CW rotation of the flagella associated with the bacteria attached to a microswimmer does increase the probability of directional change of the microswimmer, which leads to the heading bias towards the up-gradient direction.

**Figure 5:**
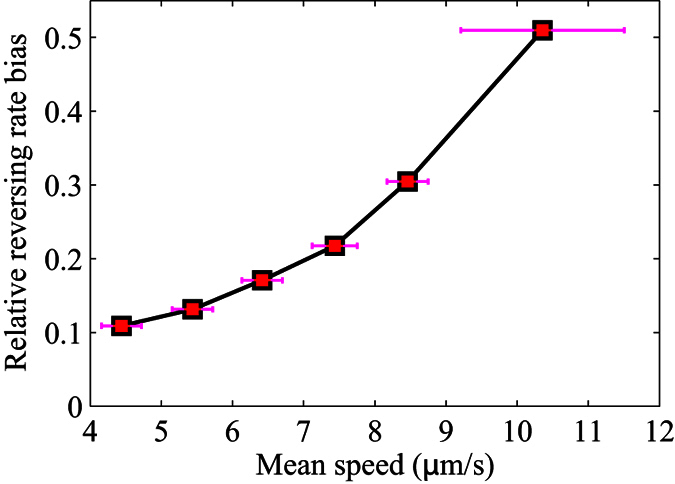
Dependence of relative reversing rate bias on mean speed. Trajectories from all five measured samples are classified into six speed intervals (4–9 and ≥9 *μ*m/s) according to their mean speeds. The horizontal data points denote the mean speeds of the trajectories that fall within each range, with the error bars indicating the standard deviations of the means.

When described in 2D, as shown in Eq. 1, the chemotactic velocity is linearly dependent on the relative reversing rate bias, which can be described by (*r*^−*y*^ −*r*^+*y*^)/(*r*^−*y*^ −*r*^+*y*^), where *r*^+*y*^ and *r*^−*y*^ are the reversing rates of the heading up and heading down cases, respectively. Figure 5 plots the relative reversing rate bias with respect to the mean speed of the trajectories, where the relative reversing rate bias grows in a superlinear fashion, although it was shown earlier that the speed bias does not contribute significantly to the drift velocity. It can be concluded that the mean speed influences the chemotactic velocity not only through being a scale factor of *V*_*C*_ as shown in Eq. 1, but also by affecting the relative reversing rate bias. Overall, the dependence of the chemotactic velocity on the mean swimming speed should be superquadratic. Since bacteria sense a spatial gradient in the form of temporal gradients as they swim through their environment, a higher translational speed usually produces a larger temporal gradient and thus, leads to improved bacterial sensing of the spatial chemical gradient.

## Discussion

Chemotaxis is a rather common and understood behavior of individual flagellated bacteria, such as *S. marcescens* and *E. coli*; it is crucial for bacteria survival because chemotaxis navigates them towards nutrient sources and away from hazardous environments. Interestingly, recent studies^27^,^28^,^29^,^30^ have observed chemotactic phenomenon in a microswimmer system driven by multiple bacteria, which implies a collective chemotactic behavior of the group of bacteria attached to a microswimmer. By statistical analysis on the swimming trajectories of the bacteria-driven microswimmers, for the first time this study elucidates the basic physical mechanisms which drive the seemingly cooperative chemotaxis among multiple bacteria attached to a common microstructure. Such chemotactic capability may prove to be beneficial for bacterial survival under certain conditions, such as when multiple bacteria are interlocked with each other, given that bacterial adhesion is ubiquitous in nature.

*S. marcescens* bacteria swim by a combination of runs and tumbles, of which the runs are directional movements with constant speeds, and the tumbles are random reorientations of the cell body with negligible displacements. However, the motion of the bio-hybrid microswimmers is characterized as a movement with relatively stable speed but incessantly changing heading direction. The propulsive forces exerted on the microswimmer at an arbitrary moment can be reduced to a force and a torque which are normally noncolinear^15^. In spite of the significant difference in the motion between the free swimming bacteria and the microswimmers, they share a similar physical model for chemotaxis in a general sense, namely, the heading direction is more persistent when moving towards higher concentrations of chemoattractant. Assuming no chemical interactions between the attached bacteria on a microswimmer, it is the flagellar rotation dynamics of the individual bacteria in response to their local chemical concentration changes that leads to the seemingly cooperative behavior of the attached bacteria during chemotaxis.

Although bacterial chemotaxis produces similar drift control in microswimmers as pH-taxis does, canonical chemotaxis differes profoundly from pH-taxis, both in the dynamics of taxis response and the sensing mechanism. As shown in this study, the speed bias of microswimmers only contributes negligibly to the chemotactic drift. However, in pH-taxis of microswimmers^31^, the speed bias contributes a substantial part of the pH-tactic drift. This divergence manifests the difference of bacterial taxis response between chemotaxis and pH-taxis. In chemotaxis, the average duration of tumble is much shorter than the average running duration, while in pH-mediated taxis, bacteria conduct prolonged tumblings^32^. The prolonged bacterial tumbling events could yield larger decrease in bacterial propulsive force when the microswimmer moves towards unfavored directions. In addition, unlike cytoplasmic-pH-mediated taxis, canonical chemotaxis is enabled by transmembrane receptor-ligand binding kinetices. The chemotaxis of bacteria-driven microswimmers indicates that bacterial physical attachment to polystyrene surfaces does not interfere with their chemotactic response. Therefore, with this study, it is reasonable to expect that any chemoattractant/repellant that is functioned by receptor-ligand binding should produce proportional chemotaxis in the multi-bacteria-driven microroots.

Differences in the measured values are seen across the five analyzed samples, as shown in Fig. 4(b–d). One source of these differences is the variances in the fabrication process between samples. For example, the mean speed of the microswimmers mainly depends on the average motility of the bacteria and the mean number of bacteria attached to a microswimmer. Since the bacterial motility and the percentage of motile bacteria are associated with the location where the bacteria are extracted from the colony^44^, slight discrepancies in the extracting location of bacteria among the samples could introduce variances in the mean speed between samples. In general, the bacterial motility parameters, such as the mean speed and the mean tumble rate, as well as the average number of bacteria attached to a microswimmer all affect the motion characteristics of the microswimmers, such as the mean speed and the heading direction reversing rate.

The results of this study not only help us better understand the chemotaxis in bio-hybrid micororobots, but also offer us some guidelines for designing and fabricating bacteria-driven microswimmers with enhanced chemotactic performance. We find that the chemotactic drift velocity increases superquadratically with the mean speed of the microswimmer. Therefore, an effective way to enhance the chemotaxis in microswimmers is to increase their mean speed, which can be achieved by various techniques, such as using bacteria with higher motility, aligning the bacteria on the microswimmer instead of random attachment, patterning the attachment location^29^ to increase the net propulsive force, and possibly decreasing the size or modifying the shape^30^ of the microswimmer to reduce the Stokes drag coefficient. On the other hand, depending on the availability or ease of deployment of a certain gradient, chemotaxis may be applied interchangeably with pH-taxis or other taxes to implement drift control in bio-hybrid microswimmers.

## Materials and Methods

### Bacteria and growth conditions

*S. marcescens* (ATCC 274, American Type Culture Collection, Manassas, VA) was first cultured in a nutrient broth (25 g Difco LB Miller Broth and 1 L deionized (DI) water, pH 7.0) at 37 °C for 3.5 h to its exponential growth phase. Then an aliquot of 2 *μ*L of the liquid culture was transfered to an agar plate (25 g Difco LB Miller Broth, 6 g Bacto Agar, 5 g glucose, 1 L de-ionized water) to grow at 30 °C for 16–20 h. After the culture period, bacteria near the leading edge of the colony was extracted and used to test the chemotactic response of free swimming bacteria or to fabricate the bio-hybrid microswimmers.

### Microswimmer fabrication

Multiple bacteria were randomly adhered to 3.1 *μ*m diameter fluorescent green polystyrene beads (*ρ* = 1.05 g/cm^3^, Fisher Scientific, Inc.) to fabricate the bio-hybrid microswimmers. To exploit the natural adherence between *S. marcescens* and the polystyrene surface, the original coating on the beads were cleaned off by a washing process, where beads were alternately sonicated in either DI water or IPA (50%) for a total of 5 cycles. The residual IPA in the bead solution was washed out with DI water, and the cleaned beads were soaked in motility buffer (0.01 M KH_2_PO_4_, 0.067 M NaCl, 10^−4^ M EDTA, pH = 7.0) to a volume percentage of 0.05%. The attachment of bacteria to the microbeads was enabled by placing an aliquot of 3 *μ*L bead solution onto the leading edge of the plate cultured bacterial colony. Previous work has shown that the motility of the microswimmers can be maximized by using bacteria from the leading edge of the colony^44^. To achieve adequate contact between bacteria and microbeads, the bead solution was gently pipetted 3–5 times to sufficiently mix the bacteria with the beads. The solution was then lifted from the plate and was incubated at room temperature for 5 minutes, allowing bacteria to attach to the beads. Subsequently, the sample was suspended in a base solution consisting of 40 *μ*L Percoll (*ρ* = 1.13 g/cm^3^, Sigma-Aldrich, St. Louis, MO) and 57 *μ*L motility buffer, where the percentage of Percoll was specifically calibrated to achieve neutral buoyancy of the microswimmers. If necessary, the solution was diluted further to achieve a concentration suitable for computer vision tracking of the microswimmers.

### Microfluidic device

A diffusion-based three-channel concentration gradient generator design^47^,^48^ was adopted to fabricate the microfluidic device used in this study for quantitative chemotaxis analysis. One significant advantage of this design is that the sample channel (middle channel) is flow-free, hence largely reducing the flow-induced forces on the bacteria and microswimmers. The concentration gradient generator was assembled from a molded hydrogel chip patterned with three microfluidic channels, namely, the source, sink and sample channel. To mold the hydrogel chip, a positive mold of the channel patterns was first fabricated by a standard soft lithography technique, during which the photoresist (SU-8 2075) was double coated to increase the height of the channels. The hydrogel chips were molded by pouring 4% (w/w) hot agarose (Eiken Chemical Co.) solution onto the silicon master mold, where each unit of the channel pattern was surrounded by a polydimethylsiloxane (PDMS) enclosure to contain the liquid agar. After the hydrogel chip was cured, it was lifted from the master mold, and the outlets of the source and sink channels were added by punching holes through the hydrogel; no outlet holes were created in the sample channel to reduce the occurrence of pressure driven flows, which constitutes a major modification to the original design of the apparatus. Subsequently the sample solution (containing bacteria or microswimmers) was loaded into the sample channel, after which the channel was covered with a coverslip, which served as the bottom surface of the channel. The assembly was finalized by sandwiching the agarose gel chip (including the diffusion section, a PDMS enclosure and a cover slip) between two prefabricated acrylic panels. Further details about the fabrication process can be found in ref. 45.

### Chemical concentration gradient

The parallel channels of the microfluidic device are separated by a layer of agarose gel, through which relatively small molecules, such as L-serine and fluorescein, diffuse with a diffusion coefficient similar to that of water^44^. Over the course of each experiment, two separate fluids were pumped through the source and sink channels at the same flow rate of 5 *μ*L/min, as shown in Fig. 1. When fluids with different chemical concentrations are flowed through the source and sink channels, a linear concentration profile develops in the sample channel at steady state, which can be determined by simple application of Fick’s Second Law. This has been corroborated in experiments^44^,^45^,^48^, where a linear intensity profile (proportional to its concentration profile at low concentrations) of fluorescein (diffusion coefficient, *D* = 4.25 × 10^−6^ cm^2^/s, at 25 °C) was observed within 20 min from the initiation of the diffusion and was maintained subsequently. Given the linear property of diffusion, L-serine (*D* = 8.8 × 10^−6 ^cm^2^/s, at 25 °C) establishes a linear steady-state concentration profile within 10 min.

### Imaging and tracking

The microfluidic device was placed under an inverted microscope (Axio Observer 100, Carl Zeiss, Oberkochen, Germany), and the samples were imaged with either a 10x (NA 0.3, fluorescence imaging of microswimmers) or 40x (NA 0.75, phase contrast imaging of bacteria) objective. Videos of the bacteria and microswimmers were captured at frames rates of 88 frames per second (fps) (FO134SB CCD, 480 × 640 pixels, Foculus) and 5 fps (QICAM, 520 × 696 pixels, QImaging), respectively. Two-dimensional (2D, *xy*-dimension) swimming trajectories of bacteria under chemotaxis was extracted and analyzed with similar methods as introduced in our previous studies^44^,^45^. A custom computer vision tracking program developed in MATLAB (R2012a, The MathWorks, Inc, Natick, MA) was used to capture the moving trajectories (2D, *xy*-dimension) of the microswimmers. In this study, the trajectories of both bacteria and microswimmers used for analysis were located far from (≥10 body lengths) any walls of the sample channel to eliminate any wall effects.

## Additional Information

**How to cite this article**: Zhuang, J. and Sitti, M. Chemotaxis of bio-hybrid multiple bacteria-driven microswimmers. *Sci. Rep.* **6**, 32135; doi: 10.1038/srep32135 (2016).

## Supplementary Material

Supplementary Information

Supplementary Video S1

Supplementary Video S2

## References

[b1] Darnton N., Turner L., Breuer K. & Berg H. C. Moving fluid with bacterial carpets. Biophys J 86, 1863–1870 (2004). 14990512 10.1016/S0006-3495(04)74253-8 PMC1304020

[b2] Tung S. & Kim J. W. Microscale hybrid devices powered by biological flagellar motors. IEEE Transactions on Automation Science and Engineering 3, 260–263 (2006).

[b3] Martel S., Tremblay C. C., Ngakeng S. & Langlois G. Controlled manipulation and actuation of micro-objects with magnetotactic bacteria. Appl Phys Lett 89, 233904 (2006).

[b4] Hiratsuka Y., Miyata M., Tada T. & Uyeda T. Q. P. A microrotary motor powered by bacteria. Proceedings of the National Academy of Sciences 103, 13618–13623 (2006). 10.1073/pnas.0604122103 PMC1564248 16950878

[b5] Behkam B. & Sitti M. Bacterial flagella-based propulsion and on/off motion control of microscale objects. Appl Phys Lett 90, 023902 (2007).

[b6] Kim M. J. & Breuer K. S. Controlled mixing in microfluidic systems using bacterial chemotaxis. Anal. Chem. 79, 955–959 (2007). 17263321 10.1021/ac0614691

[b7] Kim M. J. & Breuer K. S. Microfluidic pump powered by self-organizing bacteria. Small 4, 111–118 (2008). 18085723 10.1002/smll.200700641

[b8] Angelani L., Leonardo R. D. & Ruocco G. Self-starting micromotors in a bacterial bath. Physical Review Letters 102, 048104 (2009). 19257480 10.1103/PhysRevLett.102.048104

[b9] Sokolov A., Apodaca M. M., Grzybowski B. A. & Aranson I. S. Swimming bacteria power microscopic gears. Proc Natl Acad Sci USA 107, 969–974 (2010). 20080560 10.1073/pnas.0913015107 PMC2824308

[b10] Singh A. V. & Sitti M. Patterned and Specific Attachment of Bacteria on Biohybrid Bacteria‐Driven Microswimmers. Advanced Healthcare Materials. doi: 10.1002/adhm.201600155 (2016). 10.1002/adhm.201600155 27240122

[b11] Di Leonardo R. *et al.* Bacterial ratchet motors. Proc Natl Acad Sci USA 107, 9541–9545 (2010). 20457936 10.1073/pnas.0910426107 PMC2906854

[b12] Fernandes R., Zuniga M., Sassine F. R., Karakoy M. & Gracias D. H. Enabling cargo-carrying bacteria via surface attachment and triggered release. Small 7, 588–592 (2011). 21370460 10.1002/smll.201002036 PMC3099305

[b13] Kojima M., Zhang Z., Nakajima M. & Fukuda T. High efficiency motility of bacteria-driven liposome with raft domain binding method. Biomed Microdevices 14, 1027–1032 (2012). 23053448 10.1007/s10544-012-9711-2

[b14] Ma Q. *et al.* Construction and operation of a microrobot based on magnetotactic bacteria in a microfluidic chip. Biomicrofluidics 6, 24107–2410712 (2012). 22655018 10.1063/1.3702444 PMC3360722

[b15] Edwards M. R., Wright Carlsen R. & Sitti M. Near and far-wall effects on the three-dimensional motion of bacteria-driven microbeads. Appl Phys Lett 102, 143701 (2013).

[b16] Koumakis N., Lepore A., Maggi C. & Leonardo R. D. Targeted delivery of colloids by swimming bacteria. Nature Communications 4 (2013). 10.1038/ncomms3588 PMC4112550 24100868

[b17] Weibel D. B. *et al.* Microoxen: Microorganisms to move microscale loads. Proc Natl Acad Sci USA 102, 11963–11967 (2005). 16103369 10.1073/pnas.0505481102 PMC1189341

[b18] Xi J., Schmidt J. J. & Montemagno C. D. Self-assembled microdevices driven by muscle. Nature Materials 4, 180–184 (2005). 15654345 10.1038/nmat1308

[b19] Kim J. *et al.* Establishment of a fabrication method for a long-term actuated hybrid cell robot. Lab on a Chip 7, 1504–1508 (2007). 17960278 10.1039/b705367c

[b20] Feinberg A. W. *et al.* Muscular thin films for building actuators and powering devices. Science 317, 1366–1370 (2007). 17823347 10.1126/science.1146885

[b21] Williams B. J., Anand S. V., Rajagopalan J. & Saif M. T. A. A self-propelled biohybrid swimmer at low reynolds number. Nature Communications 5, 1–8 (2014). 10.1038/ncomms4081 24435099

[b22] Sitti M. Voyage of the microrobots. Nature 458, 1121–1122 (2009). 19407789 10.1038/4581121a

[b23] Carlsen R. W. & Sitti M. Bio-hybrid cell-based actuators for microsystems. Small 10, 3831–3851 (2014). 24895215 10.1002/smll.201400384

[b24] Steager E. B. *et al.* Electrokinetic and optical control of bacterial microrobots. J Micromech Microeng 21, 035001 (2011).

[b25] Magdanz V., Sanchez S. & Schmidt O. G. Development of a sperm-flagella driven micro-bio-robot. Advanced Materials 25, 6581–6588 (2013). 23996782 10.1002/adma.201302544

[b26] Carlsen R. W., Edwards M. R., Zhuang J., Pacoret C. & Sitti M. Magnetic steering control of multi-cellular bio-hybrid microswimmers. Lab on a Chip 14, 3850–3859 (2014). 25120224 10.1039/c4lc00707g

[b27] Traoré M., Sahari A. & Behkam B. Computational and experimental study of chemotaxis of an ensemble of bacteria attached to a microbead. Phys Rev E Stat Nonlin Soft Matter Phys 84, 1–6 (2011). 10.1103/PhysRevE.84.061908 22304117

[b28] Kim D., Liu A., Diller E. & Sitti M. Chemotactic steering of bacteria propelled microbeads. Biomed Microdevices 14, 1009–1017 (2012). 22960953 10.1007/s10544-012-9701-4

[b29] Park D. *et al.* Motility analysis of bacteria-based microrobot (bacteriobot) using chemical gradient microchamber. Biotechnology and Bioengineering 111(**1**), 134–143 (2014). 23893511 10.1002/bit.25007

[b30] Sahari A., Traore M. A., Scharf B. E. & Behkam B. Directed transport of bacteria-based drug delivery vehicles: bacterial chemotaxis dominates particle shape. Biomed Microdevices 16 **(5)**, 717–725 (2014). 24907051 10.1007/s10544-014-9876-y

[b31] Zhuang J., Carlsen R. W. & Sitti M. pH-taxis of biohybrid microsystems. Scientific Reports 5 (2015). 10.1038/srep11403 PMC4466791 26073316

[b32] Kihara M. & Macnab R. M. Cytoplasmic ph mediates ph taxis and weak-acid repellent taxis of bacteria. Journal of Bacteriology 145 **(3)**, 1209–1221 (1981). 7009572 10.1128/jb.145.3.1209-1221.1981 PMC217121

[b33] Sourjik V. & Berg H. C. Functional interactions between receptors in bacterial chemotaxis. Nature 428, 437–441 (2004). 15042093 10.1038/nature02406

[b34] Berg H. C. & Brown D. A. Chemotaxis in *Escherichia coli* analysed by three-dimensional tracking. Nature 239, 500–504 (1972). 4563019 10.1038/239500a0

[b35] Brown D. A. & Berg H. C. Temporal stimulation of chemotaxis in *Escherichia coli*. Proc. Nat. Acad. Sci. USA 71, 1388–1392 (1974). 4598304 10.1073/pnas.71.4.1388 PMC388234

[b36] Mesibov R. & Adler J. Chemotaxis toward amino acids in *Escherichia coli*. Journal of Bacteriology 112 **(1)**, 315–326 (1972). 4562400 10.1128/jb.112.1.315-326.1972 PMC251414

[b37] Dahlquist F. W., Elwell R. A. & Lovely P. S. Studies of bacterial chemotaxis in defined concentration gradients. a model for chemotaxis toward l-serine. Journal of Supramolecular Structure 4, 329–342 (1976). 772315 10.1002/jss.400040304

[b38] Ahmed T. & Stocker R. Experimental verification of the behavioral foundation of bacterial transport parameters using microfluidics. Biophysical Journal 95, 4481–4493 (2008). 18658218 10.1529/biophysj.108.134510 PMC2567943

[b39] Kalinin Y. V., Jiang L., Tu Y. & Wu M. Logarithmic sensing in *Escherichia coli* bacterial chemotaxis. Biophysical 96, 2439–2448 (2009). 10.1016/j.bpj.2008.10.027 PMC2989150 19289068

[b40] Vuppula R. R., Tirumkudulu M. S. & Venkatesh K. V. Chemotaxis of *Escherichia coli* to l-serine. Physical Biology 7, 026007 (2010). 20505226 10.1088/1478-3975/7/2/026007

[b41] Adler J. Chemoreceptors in bacteria. Science 166, 1588–1597 (1969). 4902679 10.1126/science.166.3913.1588

[b42] Clarke S. & Koshland D. E., J. Membrane receptors for aspartate and serine in bacterial chemotaxis. Journal of Biological Chemistry 254(**19**), 9695–9702 (1979). 385590

[b43] Kalinin Y., Neumann S., Sourjik V. & Wu M. Responses of escherichia coli bacteria to two opposing chemoattractant gradients depend on the chemoreceptor ratio. Journal of Bacteriology 192 **(7)**, 1796–1800 (2010). 20118262 10.1128/JB.01507-09 PMC2838042

[b44] Edwards M. R., Carlsen R. W., Zhuang J. & Sitti M. Swimming motility characterization of *Serratia marcescens*. Micro-Bio Robotics 9, 47–60 (2014).

[b45] Zhuang J. *et al.* Analytical modeling and experimental characterization of chemotaxis in serratia marcescens. Physical Review E 89, 052704 (2014). 10.1103/PhysRevE.89.052704 25353826

[b46] Rivero M. A., Tranquillo R. T., Buettner H. M. & Lauffenburger D. A. Transport mmodel for chemotactic cell-populations based on individual cell behavior. Chem. Eng. Sci. 44, 2281–2897 (1989).

[b47] Diao J. *et al.* A three-channel microfluidic device for generating static linear gradients and its application to the quantitative analysis of bacterial chemotaxis. Lab on a Chip 6, 381–388 (2006). 16511621 10.1039/b511958h

[b48] Cheng S.-Y. *et al.* A hydrogel-based microfluidic device for the studies of directed cell migration. Lab Chip 7, 763–769 (2007). 17538719 10.1039/b618463d

