# Supplementary material for: Chemotaxis of bio-hybrid multiple bacteria-driven microswimmers
Source: Sci Rep. 2016 Aug 24;6:32135. doi: 10.1038/srep32135 (PMC4995368; doi:10.1038/srep32135)
Supplement: Supplementary Information [file srep32135-s1.pdf]

Supplementary Information

Chemotaxis of bio-hybrid multiple bacteria-driven microswimmers

Jiang Zhuang[1] and Metin Sitti[1, 2,*]

[1]Department of Mechanical Engineering, Carnegie Mellon University, Pittsburgh, PA 15213, USA
[2]Max Planck Institute for Intelligent Systems, Stuttgart 70569, Germany
*Corresponding author: sitti@is.mpg.de

Supplementary Figures:

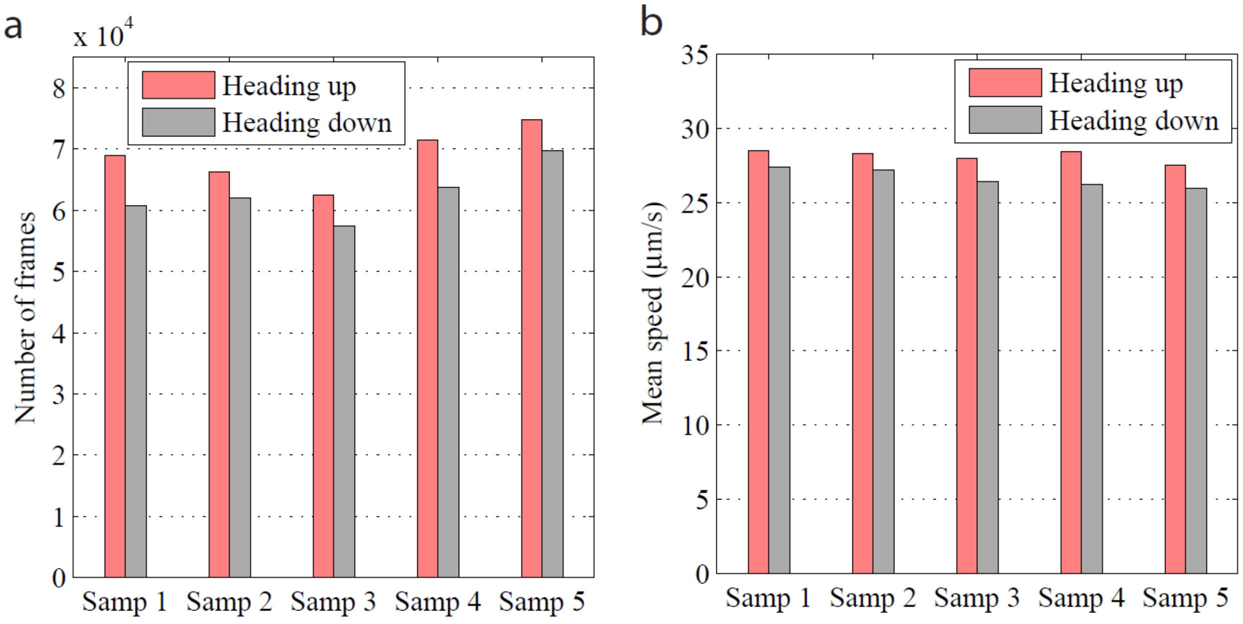

Fig. S1. Heading bias (a) and speed bias (b) of free-swimming bacteria under an L-serine gradient of 0.1 mM/mm. Five experiments were conducted on independent bacterial samples in the three-channel microfluidic concentration generator (Fig. 1 in the paper), and each sample has around 2000 bacterial swimming trajectories extracted and analyzed. The result shows that there is a consistent heading bias towards the up-gradient direction (a), where the time spent on moving towards the higher concentrations is about 10% longer than that spent on moving towards the lower concentrations. In addition, compared to the mean speed of down the gradient, the free swimming bacteria manifest a slightly higher (5% higher) speed when swimming up the chemoattractant gradient.

Supplementary Videos:

Number	Title	Description
S2	Chemotaxis of *S. marcescens* to L-serine	Phase-contrast microscopy of the bacterial distribution in the sample channel at steady state. The gradient (linear profile) is along the height dimension of the video frames, and the upside corresponds to a higher concentration of L-serine. The video is composed by multiple videos recorded at the corresponding locations along the gradient.
S3	Chemotactic drift of the bio-hybrid microrobots up the concentration gradient of L-serine	Fluorescent microscopy of the chemotactic drifting process of the microrobots driven by multiple bacteria. The gradient is along the height dimension of the video frames, and the upside corresponds to a higher concentration of L-serine.
